# Unilateral biportal endoscopic transforaminal lumbar interbody fusion versus conventional interbody fusion for the treatment of degenerative lumbar spine disease: a systematic review and meta-analysis

**DOI:** 10.1186/s12891-023-06949-y

**Published:** 2023-10-24

**Authors:** Qi Yu, Hui gen Lu, Xue kang Pan, Zhong hai Shen, Peng Ren, Xu qi Hu

**Affiliations:** 1 Bengbu Medical College, Bengbu, 233030 Anhui China; 2 Department of Orthopaedics, The Second Affiliated Hospital of Jiaxing University, Jiaxing, 314001 Zhejiang China

**Keywords:** Unilateral biportal, Endoscopy, Lumbar, Lumbar interbody fusion, Meta-analysis

## Abstract

**Background:**

This meta-analysis compares the efficacy of unilateral biportal endoscopic transforaminal lumbar interbody fusion (UBE-TLIF) to conventional interbody fusion in lumbar degenerative diseases (LDD).

**Methods:**

An extensive literature search was conducted in PubMed, Web of Science, and the Cochrane Library. Research related to UBE-TLIF published up to November 2022 was reviewed. The relevant articles were selected based on inclusion and exclusion criteria, as well as an evaluation of the quality of the data extraction literature. Meta-analysis was performed using Review Manager 5.3 software.

**Results:**

This meta-analysis included six high-quality case–control trials (CCTs) involving 621 subjects. The clinical outcomes assessment showed no statistical differences in complication rates, fusion rates, leg pain VAS scores, or ODI scores. After UBE-TLIF, low back pain VAS scores were significantly improved with less intraoperative blood loss and a shorter hospital stay. A longer time was required for UBE-TLIF, however.

**Conclusion:**

Despite the lack of sufficient high quality randomized controlled trials (RCTs) in this study, the results of this meta-analysis suggest that UBE-TLIF is more effective than open surgery in terms of length of stay, blood loss reduction during surgery, and improved low back pain after surgery. Nevertheless, the evidence will be supplemented in the future by more and better quality multicenter randomized controlled trials.

## Introduction

Lumbar degenerative disease (LDD) is common, often necessitating surgical intervention when conservative treatment fails [1–3]. Lumbar fusion surgery effectively addresses LDD by stabilizing spinal segments, restoring intervertebral disc height, relieving nerve root compression, and ensuring spinal stability [4, 5]. However, conventional open surgery presents challenges, including significant trauma, potential muscle atrophy, chronic low back pain, and adjacent segment instability. As a result, spinal surgeons have shifted their focus to minimizing surgical trauma and promoting a speedy recovery, leading to an increase in the adoption of minimally invasive transforaminal lumbar interbody fusion (MIS-TLIF) [6–8]. Nevertheless, this technique is constrained by limited operating space and a steep learning curve [9–11]. In recent years, unilateral biportal endoscopy (UBE) has emerged as a new minimally invasive spine technique. A variety of lumbar diseases have been successfully treated with UBE technology in related studies. UBE is effectively employed in addressing various lumbar spine conditions, including intervertebral disc herniation, spinal canal or intervertebral foramen stenosis decompression, and intradural cyst treatment, yielding favorable outcomes [12–14].

Heo et al. [15] reported successful cases of UBE for lumbar interbody fusion in 2017. However, uncertainties remain about the efficacy of UBE-TLIF compared to open surgery (MIS-TLIF/PLIF/TLIF). This study utilizes meta-analysis to assess the early clinical outcomes of various surgical approaches for treating lumbar degenerative diseases. The aim is to offer a more systematic and comprehensive evidence-based foundation for the clinical implementation of UBE-TLIF technology.

## Materials and methods

### Retrieval strategy

This study compared UBE-TLIF and open surgery for lumbar degenerative disease using both randomized controlled trials (RCTs) and case–control trials (CCTs). PubMed, Web of Science, and Cochrane Library databases were searched from January 2013 to November 2022, and only English articles were included. The search terms used were “biportal”, “endoscopic”, “fusion”, “interbody fusion”, and “lumbar”. To screen high-quality relevant articles, the retrieval strategy combines subject terms with free words. As each article describes “lumbar degenerative disease” differently, this is not a limitation for extending the scope of the search.

### Inclusion and exclusion criteria

Inclusion criteria: 1) Studies comparing unilateral biportal endoscopic lumbar interbody fusion and open lumbar fusion; 2) Study types included RCTs or CCTs with a minimum follow-up period of 6 months; 3) Patients of both genders diagnosed with single-segment lumbar degenerative disease based on physical examinations and imaging data (age ≥ 18 years); 4) Included studies should provide comprehensive data and include at least three of the evaluation indicators.

Exclusion criteria 1) studies without a control group; 2) studies with less than six months of follow-up; 3) studies lacking relevant data; 4) repetitive reports; 5) case reports, cadaver studies, expert opinions, technical reports, and reviews.

### Data selection

Two authors extracted data using standard extraction tables, and a third author summarized and reviewed. The data encompassed demographic characteristics, surgical information, and primary and secondary outcomes. Primary outcomes included Oswestry disability index (ODI) and visual analogue scale (VAS). Secondary outcomes included length of hospital stay, operative time, intraoperative blood loss, fusion rate, and complications. MIS-TLIF, TLIF, and PLIF were categorized under open surgery groups.

### Quality evaluation

Two authors conducted the screening based on the previously described inclusion and exclusion criteria. Methodological quality and bias risk for randomized controlled trials were assessed using the Cochrane Risk of Bias criteria [16]. Cohort studies were evaluated using the Newcastle–Ottawa Scale (NOS) [17]. Studies were scored based on population selection, comparability between groups, and outcomes, with higher scores indicating better study quality. Overall, studies were ultimately categorized as high quality (5–9 points) or low quality (0–4 points).

### Statistical analysis

ReviewManager5.3 statistical software (provided by the Cochrane collaboration network) was used for Meta analysis. Dichotomous variables were presented as odds ratios (OR), while continuous variables were expressed as weighted mean differences (WMDs) with 95% confidence intervals. Heterogeneity among study results was assessed using the I^2^ test, with a significance level set at α = 0.1. If *P* ≥ 0.1 or I^2^ ≤ 50%, homogeneity between studies existed and a fixed-effects model was used. Conversely, if *P* < 0.1 or I^2^ > 50%, the extracted data were highly heterogeneous, and sensitivity analysis was performed in order to identify the source of heterogeneity, followed by subgroup analysis. When the source of heterogeneity remains elusive, a random effects model will be employed.

## Result

### Search results and quality analysis

**Fig. 1:**
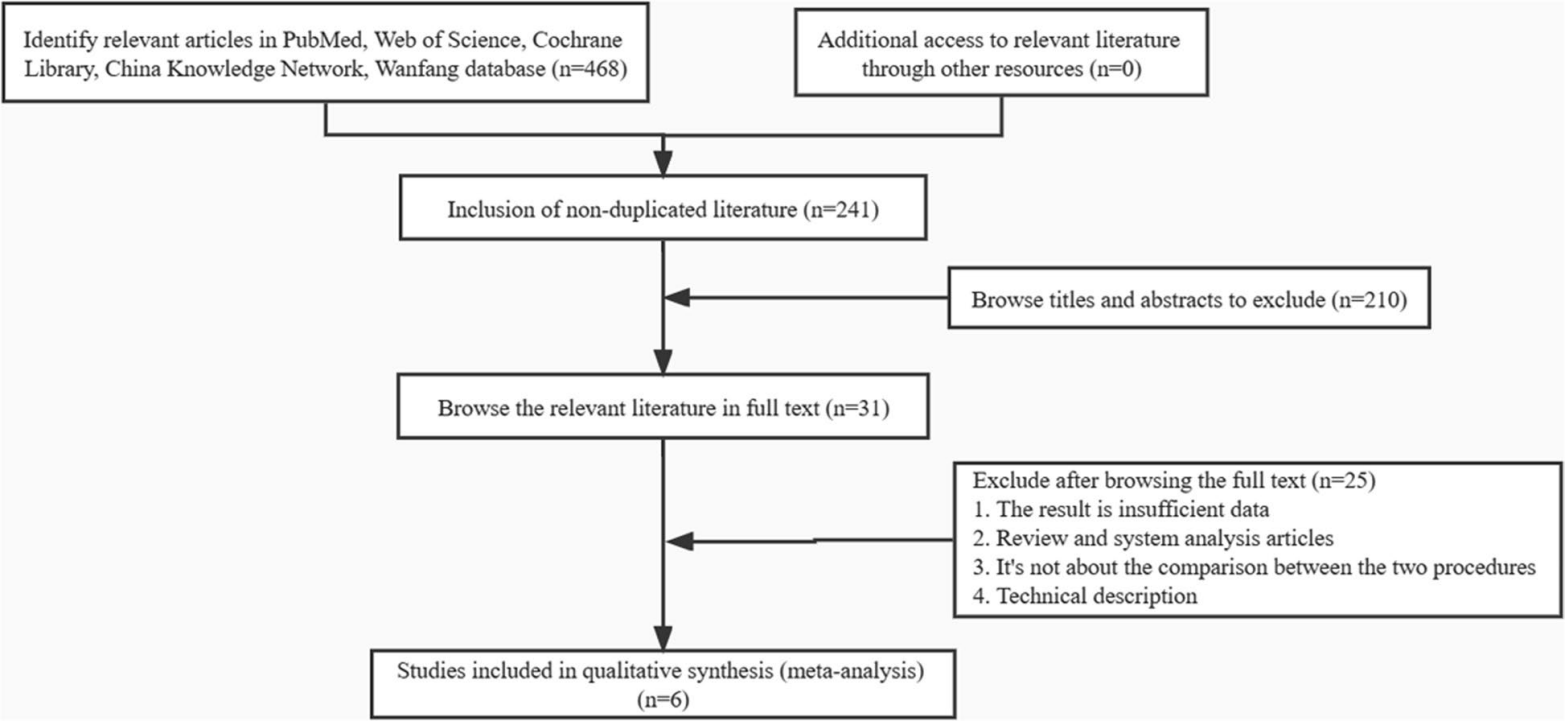
The flow chart shows the steps in the process of literature selection

Figure 1 illustrates the PRISMA evaluation procedure. Initially, 468 articles were identified through search terms, titles, and abstracts. After excluding 437 case reports, duplicate studies, or reviews, 31 articles remained that met the inclusion criteria. In the end, only six articles [18–23] were included in the meta-analysis. All studies achieved a NOS score > 5, indicating high-quality literature (Table 1).

**Table 1:** Results of quality assessment using the Newcastle–Ottawa Scale for case–control studies

Study	Selection				Comparability	Exposure			Scores
	Adequate definition of the case	Representativeness of the cases	Selection of controls	Definition of controls	Control for important factor	Ascertainment of exposure	Same method of ascertainment for cases and controls	Nonresponse rate	
Gatam et al., [18]	★		★	★	★★	★	★	★	8
Heo et al., [19]	★		★	★	★★	★	★		7
Kang et al., [20]	★	★	★	★	★★	★	★	★	9
Kim et al., [21]	★	★	★	★	★★	★	★		8
Liu et al., [22]	★		★	★	★★	★	★		7
Park et al., [23]	★		★	★	★★	★	★		7

### Demographic characteristics

The selected studies were all published between 2019 and 2022, and the pertinent demographic characteristics are detailed in Table 2. A total of 581 patients were enrolled, including 272 UBE-TLIF patients, 151 MIS-TLIF patients, and 158 PLIF/TLIF patients. In the included trials, five UBE-TLIF comparisons with MIS-TLIF and four UBE-TLIF comparisons with PLIF/TLIF were performed. The mean age of patients receiving UBE-TLIF and open surgery was 63.72 years and 62.42 years, respectively.

**Table 2:** Overview of the included studies

**Studies**	**Year published, enrollment**	**Study location**	**Study design**	**Comparison group**	**N**	**Age (y)**	**Gender M/F**	**Diagnosis**	**Fusion level**	**Follow-up (month)**
Gatam et al., [18]	2021, 2016–2020	Indonesia	CCT	UBE-TLIF	72	55.1 ± 5.12	26/46	Grade 1 or 2 DS with neurological symptoms and mechanical back pain (NSD)	L3–4(8) L4–5(56); L5–S1(8)	Overall, 12
				MIS-TLIF	73	52.3 ± 6.13	28/45		L3–4(10) L4–L5(48); L5–S1(15)	
Heo et al., [19]	2019, 2016–2017	Korea	CCT	UBE-TLIF	23	61.4 ± 9.4	7/16	DS (grade 1); IS (grade 1); central stenosis with instability; central stenosis with concomitant foraminal stenosis (NSD)	L3–4 (3); L4–5 (17); L5–S1 (3)	13.4 ± 2.5
				MIS-TLIF	46	63.5 ± 10.5	19/27		L3–4(4) L4–5(29); L5–S1(13)	
Park et al., [23]	2019 2016-NR	Korea	CCT	UBE-TLIF	71	68 ± 8	26/45	SS (7); DS (62); HNP (2)	L3-4 (13) L4-5 (50); L5-S1 (8)	17.1 ± 4.9
				TLIF	70	66 ± 9	20/50	SS (11); DS (57); HNP (2)	L3-4 (8) L4-5 (56); L5-S1 (6)	20.4 ± 7.2
Kim et al., [21]	2021, 2015–2018	Korea	CCT	UBE-TLIF	32	70.5 ± 8.26	17/15	DS (26); LS (6)	L2–L3 (1); L3–L4 (3) L4–L5(20); L5–S1 (8)	27.2 ± 5.4
				TLIF	55	67.3 ± 10.7	25/30	DS (48); LS (7)	L2–L3 (0); L3–L4 (2) L4–L5(46); L5–S1 (7)	31.5 ± 7.3
Kang et al., [20]	2021 2018–2019	Korea	CCT	UBE-TLIF	47	66.8 ± 10.41	17/30	SS with low-grade DS(NSD)	L2-3 (4); L3-4 (7) L4-5 (34); L5-S1 (20)	14.5 ± 2.3
				MIS-TLIF	32	66.38 ± 9.45	17/15		L2-3 (1); L3-4 (9) L4-5 (22); L5-S1 (11)	15.78 ± 3.16
Liu et al., [22]	2022 2020–2021	China	CCT	UBE-TLIF	27	63.89 ± 8.44	12/15	SS (7); LDH (14); LS (6)	L3/4 (4) L4/5 (18); L5/S1 (5)	11.67 ± 5.05
				PLIF	33	63.70 ± 9.69	13/20	SS (9); LDH (17); LS (7)	L3/4 (7) L4/5 (20); L5/S1 (6)	12.15 ± 4.18

*DS* degenerative spondylolisthesis, *SS* spinal stenosis, *HNP* herniated nucleus pulposus, *IS* isthmic spondylolisthesis, *LS* lytic spondylolisthesis, *NSD* no specific declaration between groups, *LDH* Lumbar disc herniation, *NR* not reported

### Primary outcome

#### Pain outcomes

**Fig. 2:**
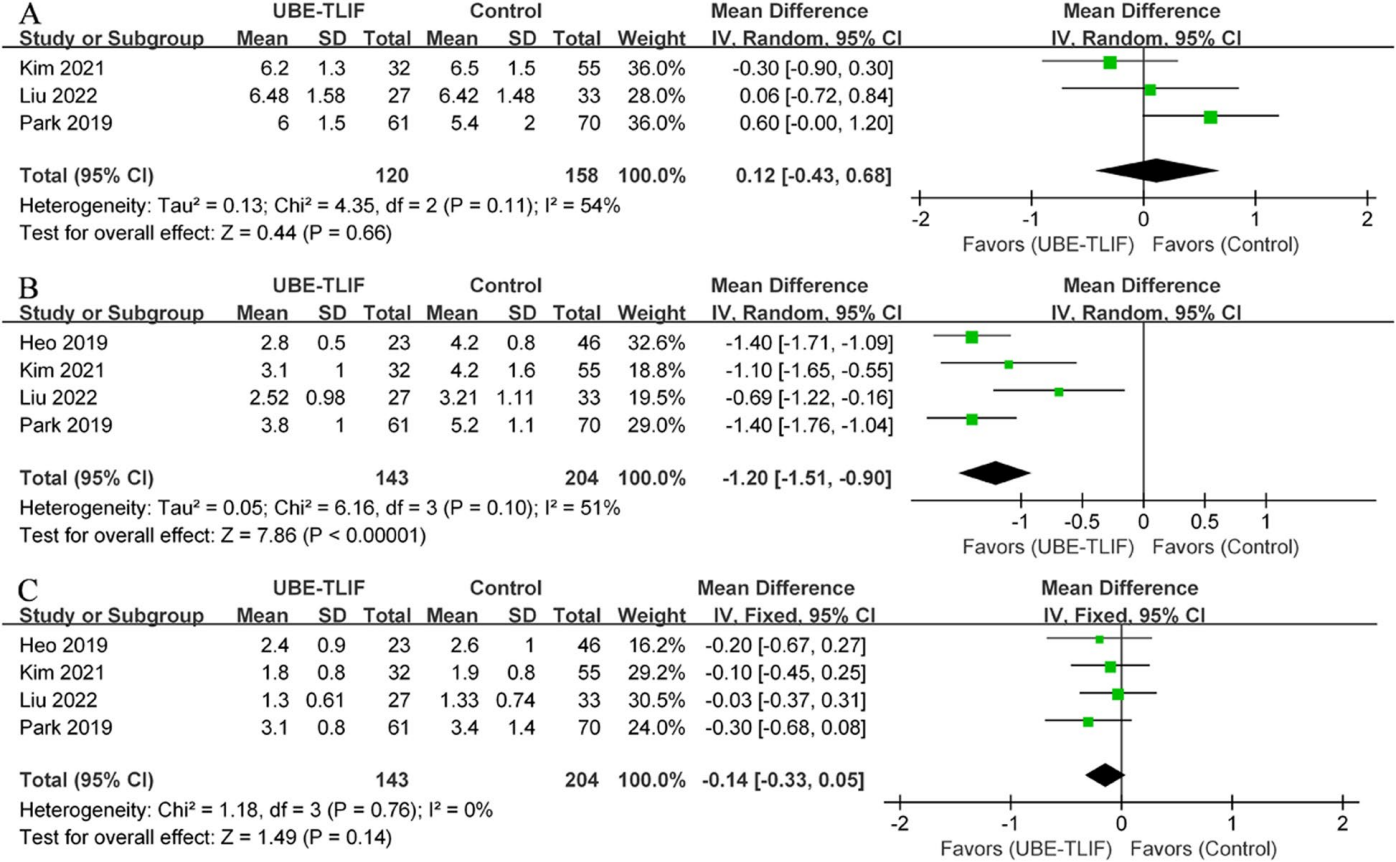
Comparison of visual analog scale for back between unilateral biportal endoscopic transforaminal lumbar interbody fusion (UBE-TLIF) and open lumbar fusion: **A** preoperative, **B** early (between 2 days and 2 weeks after) postoperative, **C** final follow-up

**Fig. 3:**
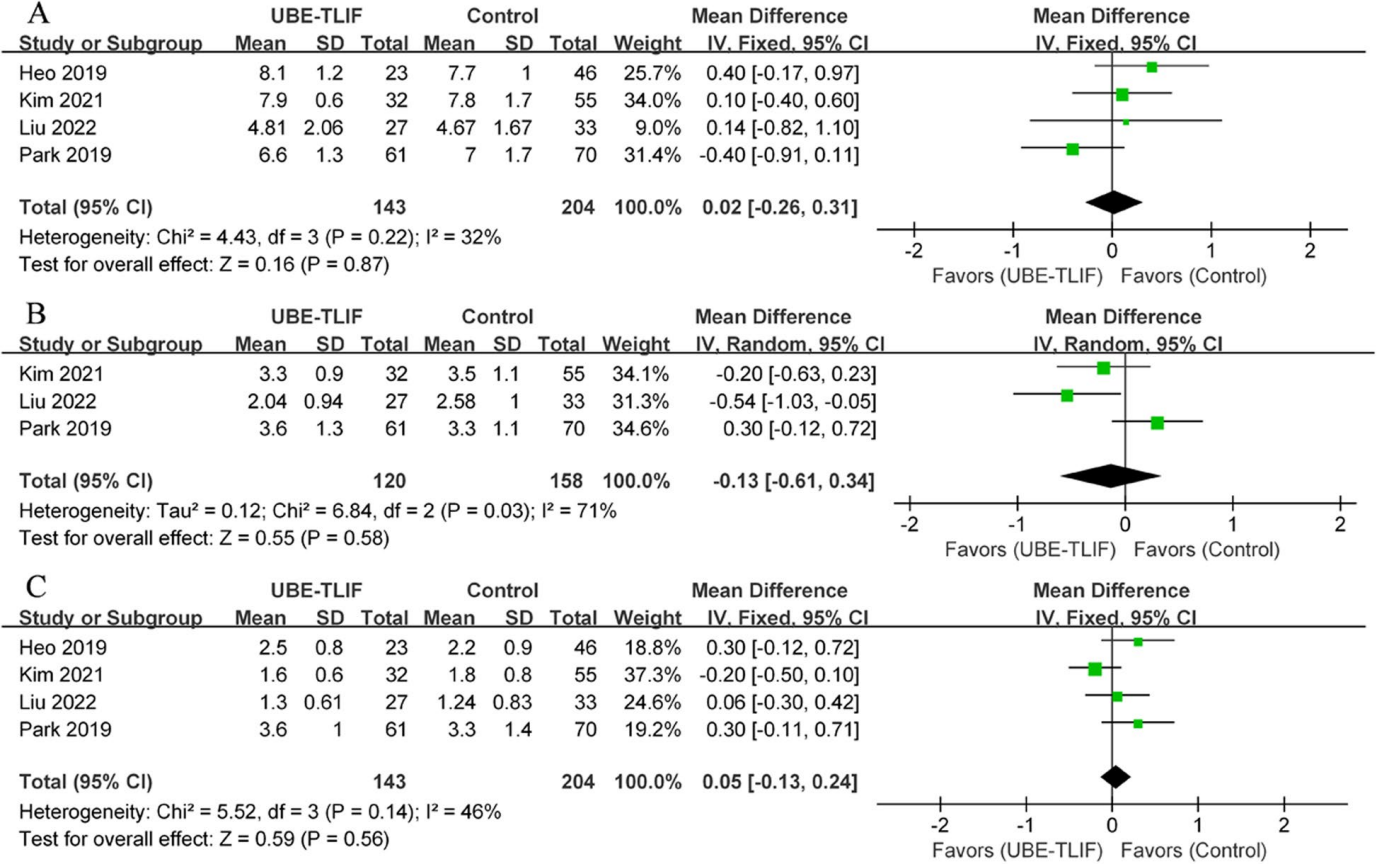
Comparison of visual analog scale for leg between unilateral biportal endoscopic transforaminal lumbar interbody fusion (UBE-TLIF) and open lumbar fusion: **A** preoperative, **B** early (between 2 days and 2 weeks after) postoperative, **C** final follow-up

Pain outcomes, assessed using VAS scores, were reported in four CCTs [19, 21–23], including UBE-TLIF (*n* = 143) and open surgery (*n* = 204). There was no statistical heterogeneity in the preoperative back VAS scores between the UBE-TLIF and open surgery groups (*P* = 0.11, I^2^ = 54%). According to the meta-analysis, there was no statistically significant difference in preoperative back VAS scores between the two groups (WMD = 0.12, 95% CI: -0.43 to 0.68, *P* = 0.66; Fig. 2A). However, the early postoperative back VAS score for the UBE-TLIF group was significantly lower than for the open surgery group (I^2^ = 51%, WMD = -1.20, 95% CI: -1.51 to -0.90, *P* < 0.00001; Fig. 2B). A meta-analysis of back pain data at the final follow-up showed no statistically significant difference between the two groups (I^2^ = 0%, WMD = -0.14, 95% CI: -0.33 to 0.05, *P* = 0.14; Fig. 2C). Regarding VAS score for leg pain, the results indicated no statistically significant difference between the two groups at each time point (preoperative: I^2^ = 32%, WMD = 0.02, 95% CI: -0.26 to 0.31, *P* = 0.87; Fig. 3A) (early postoperative period: I^2^ = 71%, WMD = -0.13, 95% CI: -0.61 to 0.34, *P* = 0.58; Fig. 3B) (final follow-up: I^2^ = 46%, WMD = 0.05, 95% CI: -0.13 to 0.24, *P* = 0.56; Fig. 3C).

#### Functional outcomes

**Fig. 4:**
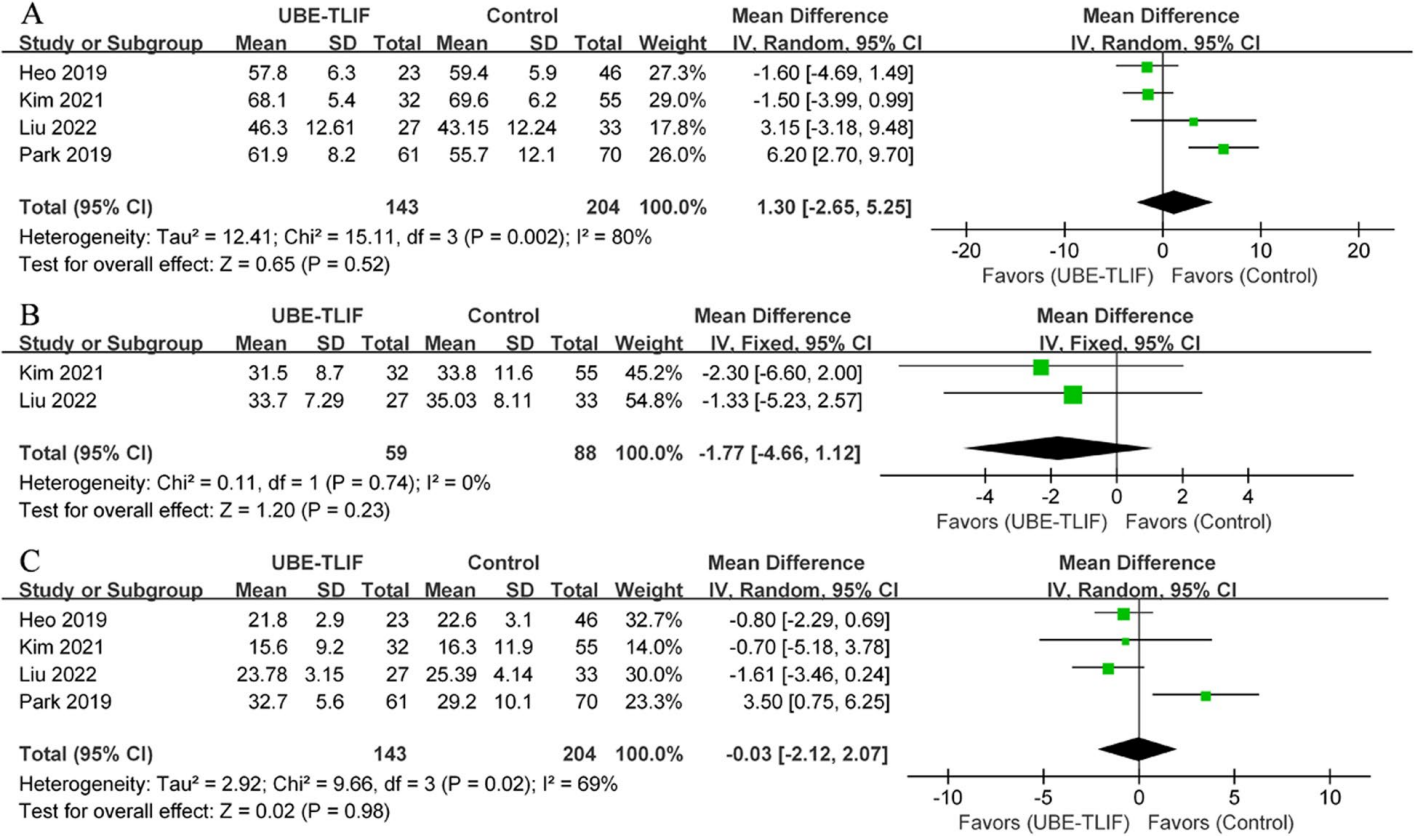
Comparison of ODI between unilateral biportal endoscopic transforaminal lumbar interbody fusion (UBE-TLIF) and open lumbar fusion: **A** preoperative, **B** early (between 2 days and 2 weeks after) postoperative, **C** final follow-up

There have been four clinical trials involving ODI [19, 21–23], including UBE-TLIF groups (*n* = 143) and open surgery groups (*n* = 204). Meta-analysis showed no statistically significant difference in ODI scores between the two groups (preoperative: I^2^ = 80%, WMD = 1.30, 95% CI: -2.65 to 5.25, *P* = 0.52; Fig. 4A) (early postoperative period: I^2^ = 0%, WMD = -1.77, 95% CI: -4.66 to 1.12, *P* = 0.23; Fig. 4B) (final follow-up: I^2^ = 69%, WMD = -0.03, 95% CI: -2.12 to 2.07, *P* = 0.98; Fig. 4C).

### Secondary outcomes

#### Operation time & hospitalization time

**Fig. 5:**
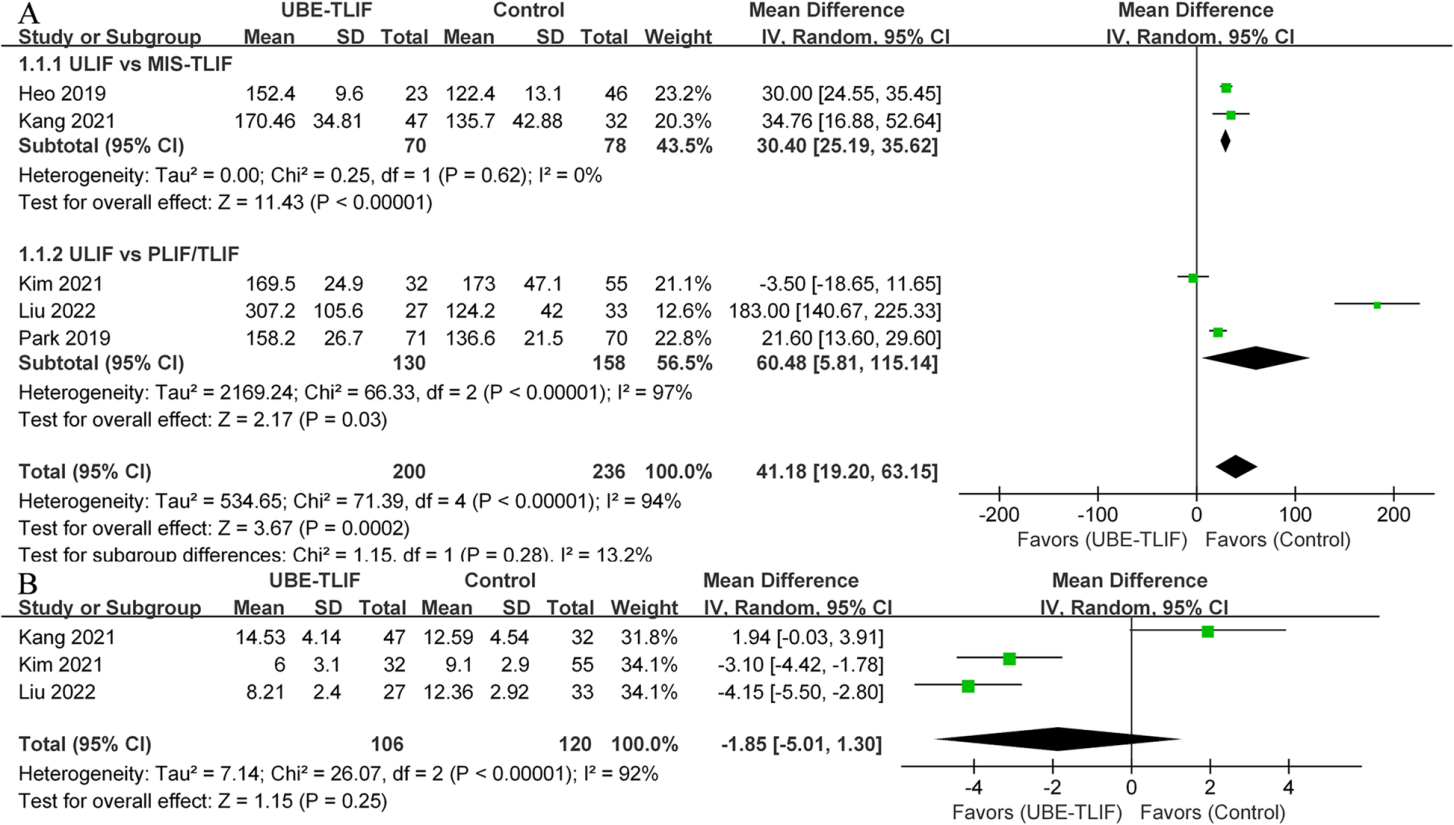
Comparison of unilateral biportal endoscopic transforaminal lumbar interbody fusion (UBE-TLIF) and open lumbar fusion: **A** operative time, **B** length of hospital stay

A total of five CCTs [19–23] reported the operative times for various approaches, including the UBE-TLIF group (*n* = 200) and the open operation group (*n* = 236). Subgroup analysis indicated significant differences in the MIS-TLIF subgroup (I^2^ = 0%, WMD = 30.40, 95% CI: 25.19 to 35.62, *P* < 0.00001) or PLIF/TLIF subgroup (I^2^ = 97%, WMD = 60.48, 95% CI: 5.81 to 115.14, *P* = 0.03). Furthermore, the combined analysis revealed a significant difference between the UBE-TLIF and open operation groups (I^2^ = 94%, WMD = 41.18, 95% CI: 19.20 to 63.15, *P* = 0.0002; Fig. 5A). Three CCTs [20–22] evaluated the length of hospital stay for both surgical approaches, including 59 patients in the UBE-TLIF group and 88 patients in the open operation group. There was no statistically significant difference between the two groups in terms of length of stay (I^2^ = 92%, WMD = -1.85, 95%CI: -5.01 to 1.30, *P* = 0.25; Fig. 5B).

#### Intraoperative blood loss & fusion rates

**Fig. 6:**
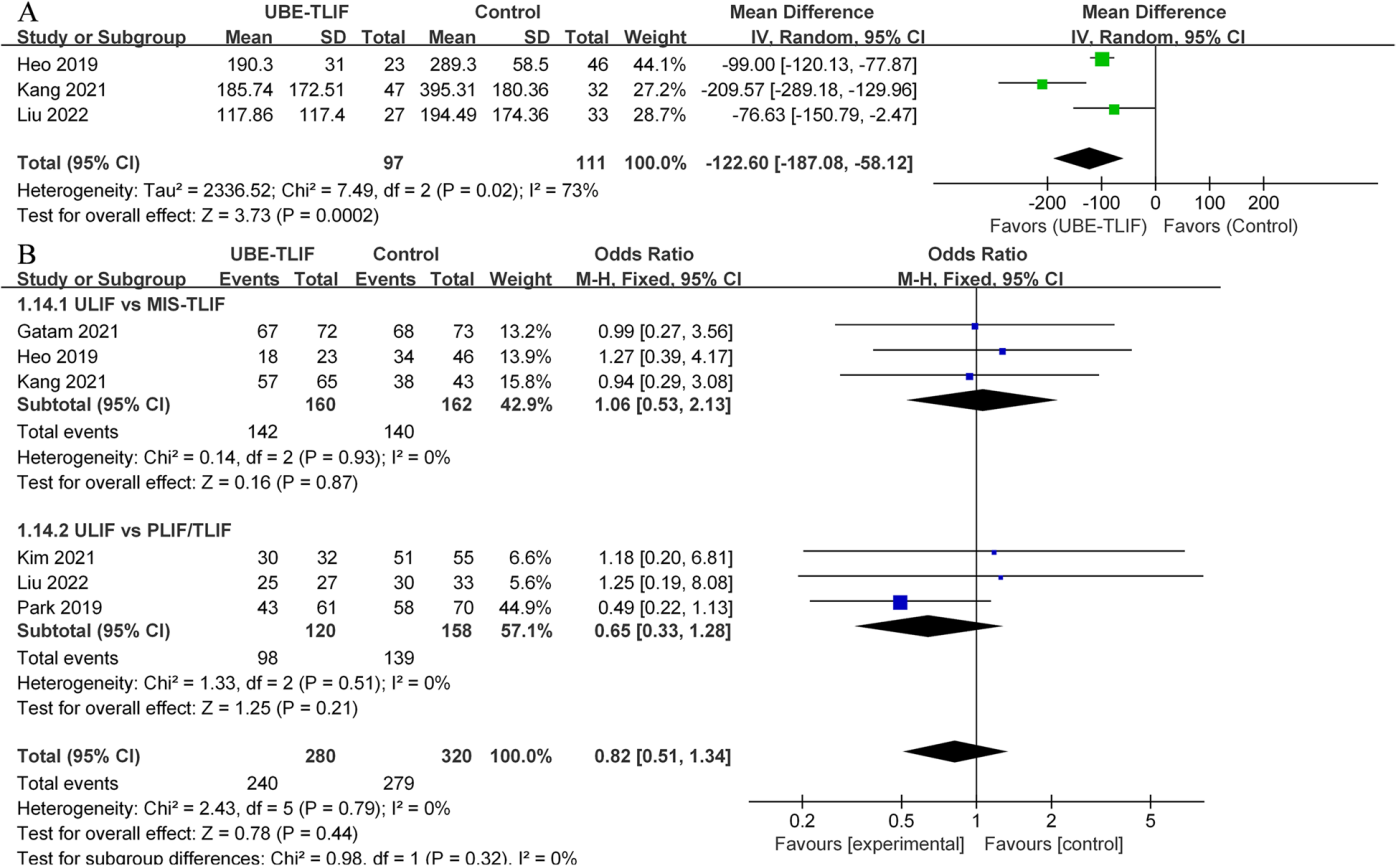
Comparison of unilateral biportal endoscopic transforaminal lumbar interbody fusion (UBE-TLIF) and open lumbar fusion: **A** estimated blood loss, **B** fusion rate

Across three retrospective studies [19, 20, 22], intraoperative blood loss was reported in two surgical approaches, including UBE-TLIF (*n* = 97) and open surgery (*n* = 111). The meta-analysis revealed significant differences between the UBE-TLIF and open operations groups (I2 = 73%, WMD = -122.60, 95% CI: -187.08 to -58.12, *P* = 0.0002; Fig. 6A). Approximately 85.7% of UBE-TLIF patients (240/280) and 87.2% of open surgery patients (279/320) reported fusion at the final follow-up in six CCTs [18–23]. There was no difference in fusion rates between MIS-TLIF subgroup (I^2^ = 0%, OR = 1.06, 95% CI: 0.53 to 2.13, *P* = 0.87) or PLIF/TLIF subgroup (I^2^ = 0%, OR = 0.65, 95% CI: 0.33 to 1.28, *P* = 0.21). Overall, the combined analysis showed no significant differences between the UBE-TLIF and open operation groups (I^2^ = 0%, OR = 0.82, 95% CI: 0.51 to 1.34, *P* = 0.44; Fig. 6B). Based on the available evidence, UBE-TLIF appears to achieve similar fusion rates to open surgery.

### Complication

**Fig. 7:**
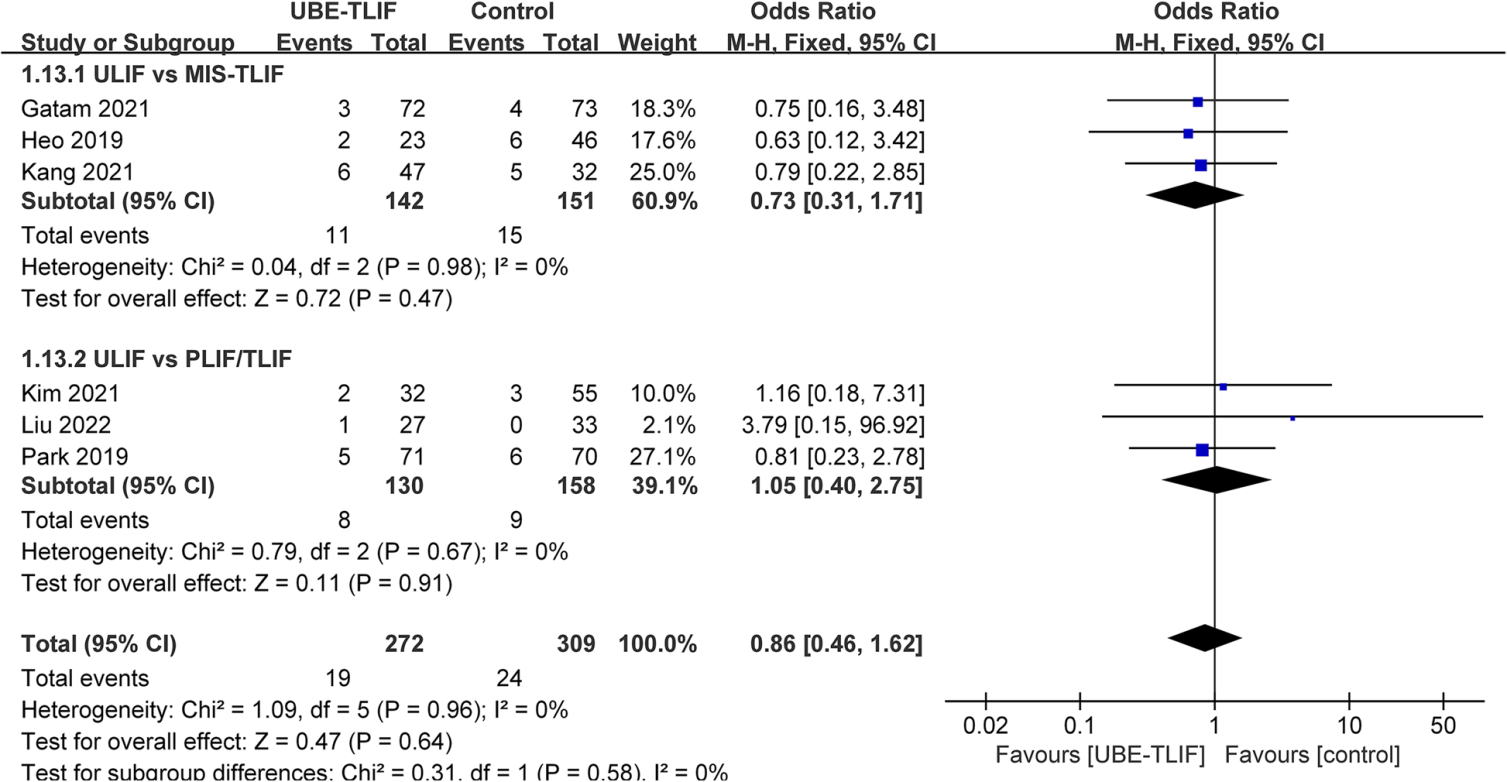
Comparison of complications between unilateral biportal endoscopic transforaminal lumbar interbody fusion (UBE-TLIF) and open lumbar fusion

**Fig. 8:**
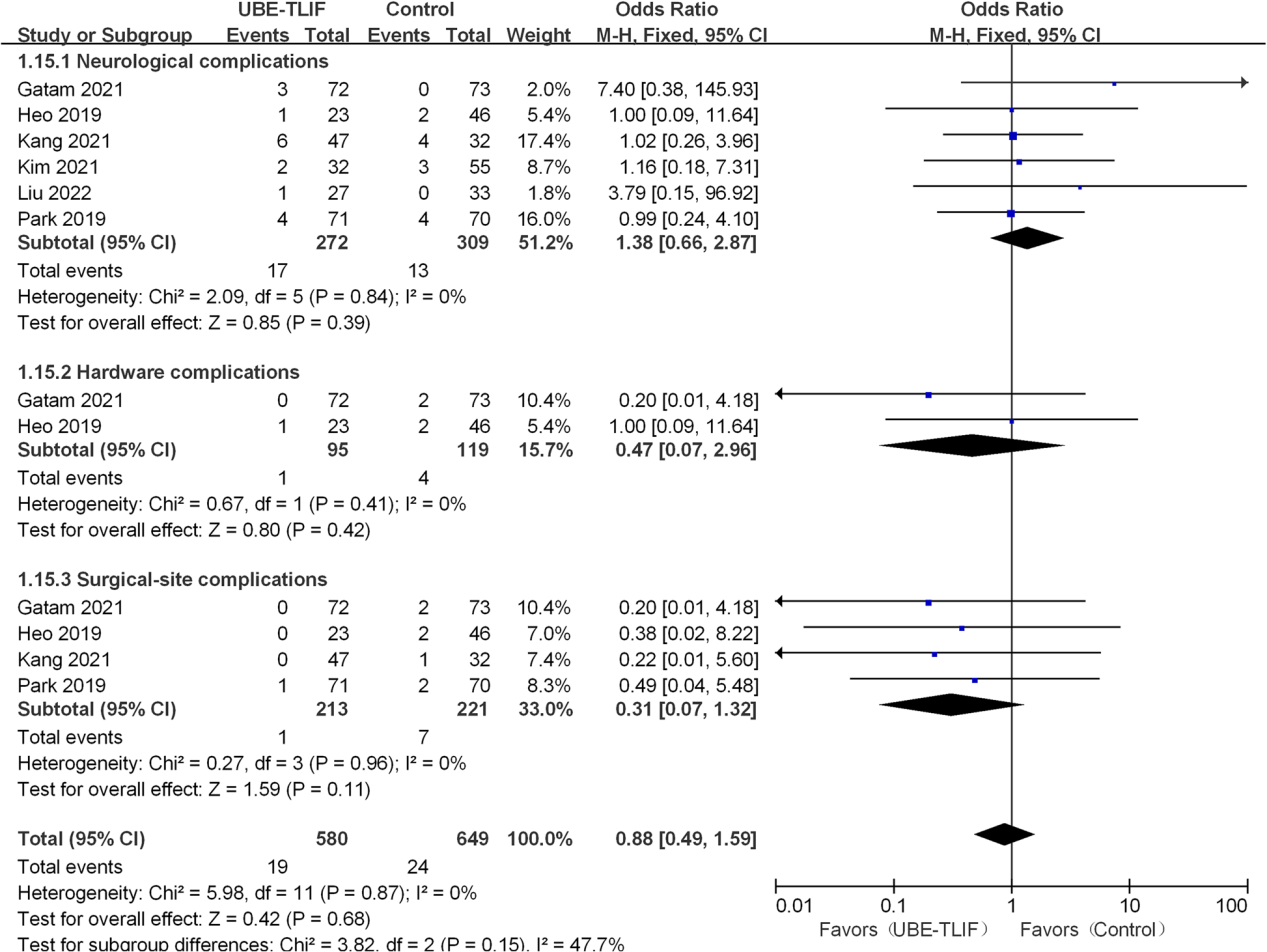
Comparison of complications rates (neurological complications, hardware complications, and surgical site complications) between minimally invasive and open lumbar fusion

All studies reported postoperative complications [18–23]. There were 19 cases (7.0%) of complications in the UBE-TLIF group, and 20 cases (6.5%) in the open operation group. Subgroup analysis shows no difference in complication rates between the MIS-TLIF subgroup (I^2^ = 0%, OR = 0.73, 95% CI: 0.31 to 1.71, *P* = 0.47) or PLIF/TLIF subgroup (I^2^ = 0%, OR = 1.05, 95% CI: 0.40 to 2.75, *P* = 0.91). Undoubtedly, the combined results indicate no significant difference between the UBE-TLIF group and the open operating group (I^2^ = 0%, OR = 0.86, 95% CI: 0.46 to 1.62, *P* = 0.64; Fig. 7). Furthermore, the postoperative complications can be classified into three categories: neurological complications (postoperative epidural hematoma, dural tear, incomplete decompression, transient palsy, nerve root injury), hardware-related complications (cage subsidence), and surgical site complications (postoperative infection, DVT). Meta-analysis results showed no statistically significant complications in any subgroup (Fig. 8).

### Publication bias

This study identified high heterogeneity in postoperative leg VAS scores, operation time, preoperative and postoperative ODI scores, length of hospital stay, and intraoperative blood loss. The duration of stay was determined through a leave-one-out sensitivity analysis. Only after omitting Kang et al. [20] was a shortened postoperative hospital stay in the UBE-TLIF group observed. Sensitivity analysis indicated minimal heterogeneity in other indicators, affirming result reliability. The author suggests that heterogeneity could stem from cultural disparities, variations in surgeon skill levels, differences in postoperative care protocols, and the presence of complications. In assessing publication bias, an asymmetric funnel plot suggests potential bias in reporting postoperative complications.

## Discussion

### Necessity analysis

Advances in medical devices and surgical concepts have made minimally invasive surgery the mainstay of treatment for LDD. The fundamental objective of minimally invasive surgery is to alleviate symptoms [24]. In contrast to conventional open surgery, UBE-TLIF instruments are positioned within two distinct channels, affording greater maneuverability to achieve more comprehensive decompression. Endoscopes were employed to facilitate visual assessment of procedures encompassing endplate manipulation, cartilage endplate removal, and bony endplate exposure. Advocates of the UBE-TLIF technique emphasize multiple merits, including minimized incision size, less strain on paraspinal muscles, and a shortened learning curve [23, 25–27]. Consequently, given the lack of systematic reviews, this meta-analysis was undertaken to establish a more reliable foundation for clinical decision-making.

### Clinical efficacy evaluation

According to Fairbank et al. [28], the ODI and VAS scores effectively gauge the impact of low back pain on daily functional capacity. In the clinical data of this study, there was no significant difference in fusion rate, leg VAS score, or ODI improvement between UBE-TLIF and open surgery. In conclusion, UBE-TLIF has not demonstrated a higher fusion rate or greater functional improvement compared with open surgery. Compared with the open surgery group, the early low back VAS score of the UBE-TLIF group decreased by 1.20 units. Although open surgery remains the prevailing treatment approach for LDD, the combined early postoperative low back VAS scores in this study favor UBE-TLIF. There is no doubt that early improvement in low back pain can be attributed to the minimally invasive nature of UBE-TLIF surgery and its preservation of spinal column anatomy. Furthermore, the percutaneous tension-free access incision utilized in UBE-TLIF surgery mitigates soft tissue compression and stripping, thereby reducing the occurrence of chronic back pain [29]. There has been evidence that postoperative low back pain is associated with muscle denervation and atrophy, emphasizing the importance of reducing muscle damage during surgery [23, 30].

Liu et al. [22] and Kang et al. [20] reported increased early postoperative serum creatine phosphokinase (CPK) and C-reactive protein (CRP) levels in both UBE-TLIF and PLIF groups, with a smaller rise in the UBE-TLIF group. According to the above study, UBE-TLIF surgery reduces systemic inflammation, medication-induced muscle damage, postoperative pain, and improves quality of life. Meta-analysis also showed that UBE-TLIF had less intraoperative blood loss but a longer operation time (*P* < 0.05), which is consistent with the results of previous studies [19–23]. The author speculates that surgeon experience is the main factor behind this difference in operative time. Spine surgeons who are unfamiliar with endoscopic manipulation may find UBE-TLIF challenging because of the need to manipulate biportal channels and optimize the visibility of the surgical field. Additionally, the learning curve of a new technique can affect the operative time. In a 2020 study, Kim et al. [31] reported that approximately 34 cases were needed to attain proficiency in the UBE-TLIF technique. Wang et al. [32] demonstrate that the operation time of UBE-TLIF was gradually shortened with the increase in the number of surgical cases, ultimately stabilizing after the completion of 17 instances.

The initial learning curve during surgery can impact the occurrence of postoperative complications, especially neurological issues such as dural tears, nerve root injuries, and epidural hematomas. Neurological complications were 1.38 units higher in the UBE-TLIF group than in the open surgery group, with dural tears leading the way, although there was no statistically significant difference between the two groups. Dural tears may manifest early during the learning process or be linked to adhesion of the ligamentum flavum. Smaller dural tears can typically be repaired with gelatin sponge, while larger tears usually necessitate conversion to MIS-TLIF. There were no cases of large dural tears requiring open surgery in the included studies.

### Limitation analysis

The UBE-TLIF technique can theoretically avoid extensive muscle stripping, relieve low back pain, encourage patients to move early, and reduce the risk of complications caused by long-term bed rest. It is especially suitable for patients with poor basic conditions. Furthermore, UBE-TLIF is associated with shorter hospital stays, supporting rehabilitation and reducing hospitalization costs. However, this meta-analysis has several limitations: 1) Due to the recent introduction of UBE-TLIF, there is limited available literature; 2) Despite the overall high quality of the included studies, the lack of RCTs may have influenced the findings; 3) Variations in follow-up duration among the included studies may influence outcomes; 4) The lack of distinction between different surgical procedures may introduce bias when describing them as open procedures.

## Conclusions

The meta-analysis results indicate that UBE-TLIF outperforms open surgery in reducing hospital stay, decreasing intraoperative blood loss, and improving early postoperative function. However, no significant difference was found in long-term function between the two groups. Nevertheless, additional high-quality multicenter randomized controlled trials are required to bolster these results.

## Data Availability

All data generated or analysed during this study are included in this published article [and its supplementary information files].

## Abbreviations

LDD: Lumbar degenerative diseases
UBE-TLIF: Unilateral biportal endoscopic transforaminal lumbar interbody fusion
CCTs: Case–control trials
RCTs: Randomized controlled trials
UBE: Unilateral biportal endoscopy
MIS-TLIF: Minimally invasive transforaminal lumbar interbody fusion
VAS: Visual analog scale
ODI: Oswestry Disability Index
NOS: Newcastle–Ottawa scale
CPK: Creatine phosphokinase
CRP: C-reactive protein
